# Acute Transverse Myelitis Secondary to Severe Acute Respiratory Syndrome Coronavirus 2 (SARS-CoV-2): A Case Report

**DOI:** 10.5811/cpcem.2020.6.48462

**Published:** 2020-06-22

**Authors:** Muhammad Durrani, Kevin Kucharski, Zachary Smith, Stephanie Fien

**Affiliations:** Inspira Medical Center, Department of Emergency Medicine, Vineland, New Jersey

**Keywords:** Acute Transverse Myelitis, Severe Acute Respiratory Syndrome Coronavirus 2 (SARS-CoV-2), Para-infectious Acute Transverse Myelitis, Post-infectious Acute Transverse Myelitis

## Abstract

**Introduction:**

Respiratory viral illnesses are associated with diverse neurological complications, including acute transverse myelitis (ATM). Among the respiratory viral pathogens, the Coronaviridae family and its genera coronaviruses have been implicated as having neurotropic and neuroinvasive capabilities in human hosts.^1^ Despite previous strains of coronaviruses exhibiting neurotropic and neuroinvasive capabilities, little is known about the novel severe acute respiratory syndrome coronavirus 2 (SARS-CoV-2) and its involvement with the central nervous system (CNS). The current pandemic has highlighted the diverse clinical presentation of SARS-CoV-2 including a possible link to CNS manifestation with disease processes such as Guillain-Barré syndrome and cerebrovascular disease. It is critical to shed light on the varied neurological manifestation of SARS-CoV-2 to ensure clinicians do not overlook at-risk patient populations and are able to provide targeted therapies appropriately.

**Case Report:**

While there are currently no published reports on post-infectious ATM secondary to SARS-CoV-2, there is one report of parainfectious ATM attributed to SARS-CoV-2 in pre-print. Here, we present a case of infectious ATM attributed to SARS-CoV-2 in a 24-year-old male who presented with bilateral lower-extremity weakness and overflow urinary incontinence after confirmed SARS-CoV-2 infection. Magnetic resonance imaging revealed non-enhancing T2-weighted hyperintense signal abnormalities spanning from the seventh through the twelfth thoracic level consistent with acute myelitis.

**Conclusion:**

The patient underwent further workup and treatment with intravenous corticosteroids with improvement of symptoms and a discharge diagnosis of ATM secondary to SARS-CoV-2.

## INTRODUCTION

Transverse myelitis is typified by an acute or subacute inflammatory myelopathy resulting in potentially disabling neurological deficits such as motor weakness and sensory deficits, as well as autonomic dysfunction. Acute transverse myelitis (ATM) has been associated with a variety of different etiologies, which have been subdivided into compressive and non-compressive myelopathies. Non-compressive etiologies include infectious, autoimmune, ischemic, paraneoplastic, radiation effects, post-vaccination, and post-infectious, as well as idiopathic causes. The diagnosis of ATM, while challenging for the clinician, is important to recognize as it is associated with significant morbidity and mortality leaving two-thirds of infected patients with moderate to severe permanent disability.^2^–^4^

The incidence rate of ATM has been estimated to be between one and eight new cases per million annually, but recent data show that it may be as high as 31 cases per million.^5^ While this disease entity can occur at any age, the mean age of onset ranges from 35–40.^6^ Most studies conclude that men and women are affected equally, although some studies do show a female predominance.^6^ Diagnosis is based upon uniform diagnostic criteria published by the Transverse Myelitis Consortium Working Group.^7^ These criteria rely on the exclusion of extra-axial compressive etiology by neuroimaging along with inclusion of sensory, motor, or autonomic dysfunction attributed to the spinal cord, bilateral signs/symptoms, as well as a clearly defined sensory level with demonstration of inflammation within the spinal cord through either cerebrospinal fluid (CSF) or magnetic resonance imaging (MRI) studies.^6^ Treatment is varied and depends on the accurate identification of etiology to guide treatment protocols.

## CASE REPORT

A 24-year-old male with no significant medical history presented to the emergency department (ED) with complaint of fever and chills, along with nausea and non-bloody, non-bilious vomiting. He denied recent travel or sick contacts. He was febrile and tachypneic with findings of patchy airspace disease throughout both lungs compatible with multifocal pneumonia on computed tomography of the chest without contrast. He was subsequently admitted to the hospital for three days. Nasopharyngeal swabs were positive for severe acute respiratory syndrome coronavirus 2 (SARS-CoV-2) on reverse-transcriptase polymerase chain reaction (RT-PCR). He was treated with supportive care and demonstrated clinical improvement.

His respiratory pathogen panel did not reveal any coinfection and his legionella antigen, blood cultures, respiratory cultures, and human immunodeficiency virus (HIV) testing were also negative. He was discharged home but subsequently presented to the ED nine days later with symptoms of bilateral lower-extremity weakness in addition to developing overflow urinary incontinence. He denied any history of trauma, pain, or similar symptoms in the past. Upon arrival, his vitals revealed a blood pressure of 111/61 millimeters of mercury, pulse 97 beats per minute, respiratory rate 16 breaths per minute, 98% oxygen saturation on room air, and temperature of 37.3° Celsius.

His neurological examination revealed bilaterally absent knee and ankle reflexes with equivocal plantar reflexes, and flaccid, lower-extremity paraplegia bilaterally, in addition to evidence of overflow urinary incontinence. His lower-extremity sensory examination and anal tone were normal. His physical examination was otherwise normal. Laboratory workup included a complete blood count, complete metabolic panel, thyroid testing, inflammatory markers, repeat nasopharyngeal RT-PCR SARS-CoV-2 testing, and urinalysis, which were found to be unexceptional with a negative SARS-CoV-2 result on hospital day one. The patient underwent MRI of the spine as well as a lumbar puncture (LP). The MRI showed a non-enhancing T2-weighted hyperintense signal abnormality spanning from the seventh through the twelfth thoracic level consistent with acute myelitis (Image).

**CPC-EM Capsule**

What do we already know about this clinical entity?

Transverse myelitis is a focal inflammatory myelopathy causing motor, sensory, and autonomic dysfunction. Diagnosis rests upon clinical findings as well as serologic, magentic resonant imaging, and cerebral spinal fluid studies.

What makes this presentation of disease reportable?

Coronaviridae have been shown to have neurotropic and neuro-invasive capabilities, yet little is known about severe acute respiratory coronavirus 2 (SARS-CoV-2). We present the second case of acute myelitis attributed to SARS-CoV-2.

What is the major learning point?

SARS-CoV-2 is associated with a variety of neurological manifestations, including myelitis. Diagnosis should utilize established diagnostic criteria.

How might this improve emergency medicine practice?

Reinforcing the unique presentations of SARS-CoV-2 and myelitis yields a better understanding of the disease entities, allowing a focused investigation for accurate diagnosis and treatment.

CSF studies from the LP were consistent with a lymphocytic pleocytosis, normal glucose and protein levels, and electrophoresis. The patient underwent further workup to rule out other causes of transverse myelitis with CSF immunoglobulin G index, CSF-specific oligoclonal bands, aquaporin-4 antibodies, B-12 level, methylmalonic acid level, as well as a workup for HIV, other infectious diseases, autoimmune disease, connective tissue disease, and multiple sclerosis. He was diagnosed with post-infectious myelitis secondary to SARS-CoV-2 infection. Treatment was initiated with intravenous (IV) methylprednisolone and supportive care with noted improvement in bilateral lower-extremity strength. Interestingly, repeat SARS-CoV-2 testing was done on hospital day four, which returned positive. Hence, it is difficult to ascertain whether the patient demonstrated post-infectious ATM as opposed to parainfectious ATM secondary to SARS-CoV-2.

## DISCUSSION

ATM is characterized by focal inflammation of the spinal cord leading to varied severity of motor, sensory, and autonomic dysfunction. Although uncommon, it is paramount to distinguish it from other neurologic etiologies due to its potential for permanent disability. The diagnosis is based on characteristic clinical findings in addition to serologic, MRI, and CSF studies. As previously noted, the *Coronaviridae* family and its genera coronaviruses have been implicated as having neurotropic and neuroinvasive capabilities in human hosts.^1^ They have been associated with the development of neuropsychiatric symptoms, seizure activity, encephalomyelitis, acute flaccid paralysis, and Guillain-Barré syndrome, as well as cerebrovascular disease.^1^,^8^

Previous studies in mice have proposed that human coronavirus may reach the CNS via the olfactory bulbs, as viral antigens were initially detected there followed by propagation and detection in whole brain tissue days later.^1^,^9^ Subsequent viral infection of CNS glial and neuronal cells triggers demyelination as well as an inflammatory response.^1^ Other pathways proposed for viral entry have implicated both hematogenous spread as well as a retrograde axonal transport pathway for entry into the CNS.^10^,^11^

Recently, there has been a growing body of evidence supporting the association of SARS-CoV-2 with neurological abnormalities. A systematic review looking at the incidence of secondary neurological disease in patients diagnosed with SARS-CoV-2 found rates to vary from 6–36.4%.^11^ Additionally, the first case report of acute infectious myelitis associated with concurrent SARS-CoV-2 was only recently described.^12^ Here, we present the second case of acute myelitis attributed to SARS-CoV-2 infection. Considering the chronological association of a confirmed positive SARS-CoV-2 infection and the development of signs and symptoms consistent with ATM nine days later, we speculate that SARS-CoV-2 may have played a role in the development of ATM in this patient.

During his workup for ATM, this patient tested negative on repeat SARS-CoV-2 testing on hospital day one but tested positive for SARS-CoV-2 on hospital day four. Hence, it is difficult to ascertain whether the patient demonstrated post-infectious ATM as opposed to parainfectious ATM secondary to SARS-CoV-2. The diagnosis of parainfectious or post-infectious ATM relies upon a stepwise approach to rule out compressive etiologies as well as other inflammatory and non-inflammatory etiologies of ATM (Figure).

Our patient met the inclusion criteria for diagnosis of ATM based on bilateral motor symptoms and autonomic dysfunction with bladder incontinence along with evidence of CSF lymphocytic pleocytosis and characteristic MRI findings while ruling out other infectious, autoimmune, and connective tissue etiologies. Treatment of ATM must be individualized to the patient and underlying etiology that may have caused ATM. There are currently no established regimens for treatment of SARS-CoV-2 post-infectious or parainfectious transverse myelitis. Treatment for other infectious-mediated ATM include antivirals, antibiotics, corticosteroids, and IV immunoglobulin, but their efficacy has yet to be completely defined. Overall, a single case report is not robust enough to suggest a definitive link between ATM and SARS-CoV-2. More research and case reports are necessary to support a causal relationship. Despite this, clinicians must be aware of the possibility of an association with SARS-CoV-2 and be aware of the salient features of ATM for early diagnosis, workup, and potential treatment to prevent permanent disability.

## CONCLUSION

In summary, we hypothesize that this patient’s ATM was precipitated by SARS-CoV-2 leading to a diagnosis of post-infectious or parainfectious ATM. ATM has a varied presentation and is associated with significant morbidity and mortality that necessitates increased awareness and vigilance on part of the clinician. This article is the second reported case of ATM attributed to SARS-CoV-2 infection, and should serve to reinforce the unique presentations of SARS-CoV-2 and ATM.

## Figures and Tables

**Figure:**
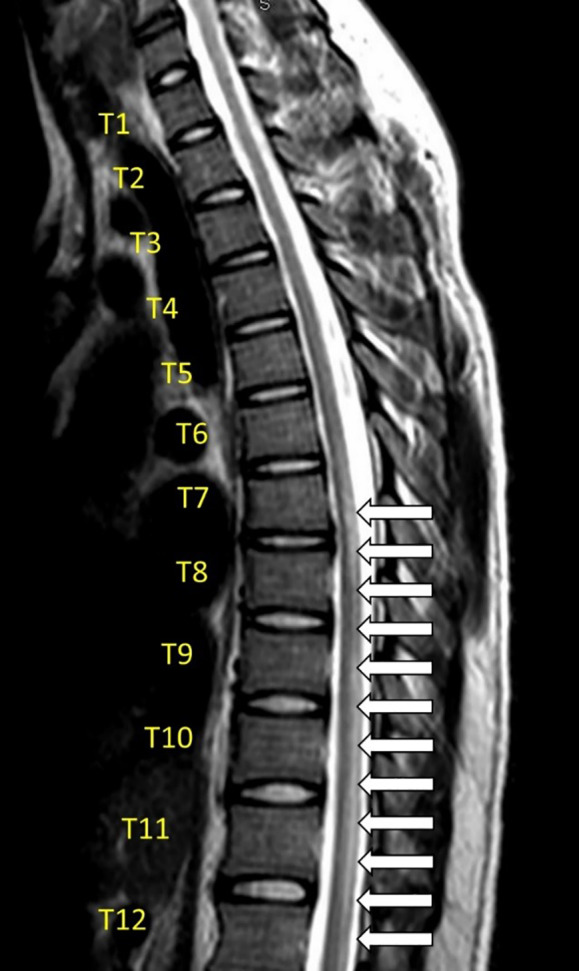
Summary of acute transverse myelitis and proposed diagnostic workup of post-infectious myelitis.

**Image:**
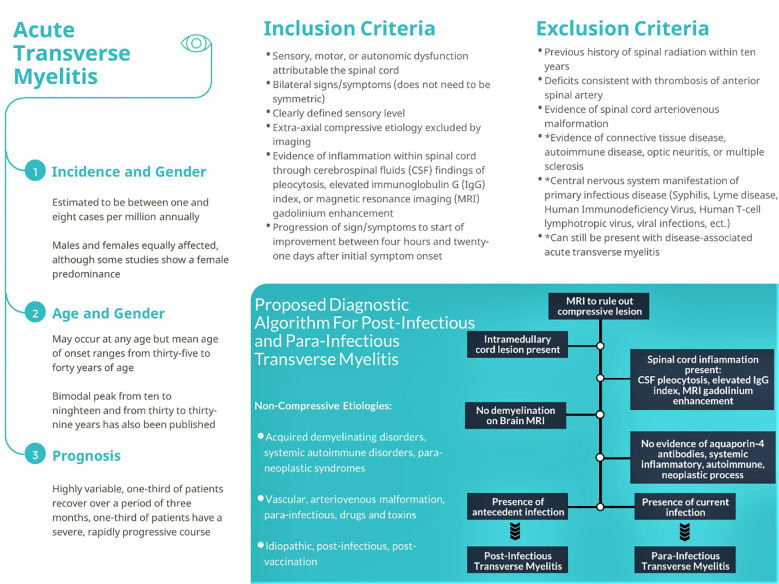
T2 sagittal image of thoracic spine showing hyperintensity in the spinal cord from the seventh through the twelfth thoracic level suggestive of transverse myelitis (arrows).
